# Reactive Oxygen Species Generated by NADPH Oxidases Promote Radicle Protrusion and Root Elongation during Rice Seed Germination

**DOI:** 10.3390/ijms18010110

**Published:** 2017-01-13

**Authors:** Wen-Yan Li, Bing-Xian Chen, Zhong-Jian Chen, Yin-Tao Gao, Zhuang Chen, Jun Liu

**Affiliations:** Argo-Biological Gene Research Center, Guangdong Academy of Agricultural Sciences, Guangzhou 510640, China; liwy1023@foxmail.com (W.-Y.L.); bxchen@agrogene.ac.cn (B.-X.C.); chenzhongjian@agrogene.ac.cn (Z.-J.C.); gaoyintao@agrogene.ac.cn (Y.-T.G.); chenzhuang@agrogene.ac.cn (Z.C.)

**Keywords:** seed germination, NADPH oxidases, reactive oxygen species (ROS), *Oryza sativa*, radicle and root elongation, gene expression

## Abstract

Seed germination is a complicated biological process that requires regulation through various enzymatic and non-enzymatic mechanisms. Although it has been recognized that reactive oxygen species (ROS) regulate radicle emergence and root elongation in a non-enzymatic manner during dicot seed germination, the role of ROS in monocot seed germination remains unknown. NADPH oxidases (NOXs) are the major ROS producers in plants; however, whether and how NOXs regulate rice seed germination through ROS generation remains unclear. Here, we report that diphenyleneiodinium (DPI), a specific NOX inhibitor, potently inhibited embryo and seedling growth—especially that of the radicle and of root elongation—in a dose-dependent manner. Notably, the DPI-mediated inhibition of radicle and root growth could be eliminated by transferring seedlings from DPI to water. Furthermore, ROS production/accumulation during rice seed germination was quantified via histochemistry. Superoxide radicals (O_2_^−^), hydrogen peroxide (H_2_O_2_) and hydroxyl radicals (^•^OH) accumulated steadily in the coleorhiza, radicle and seedling root of germinating rice seeds. Expression profiles of the nine typical NOX genes were also investigated. According to quantitative PCR, *OsNOX5*, *7* and *9* were expressed relatively higher. When seeds were incubated in water, *OsNOX5* expression progressively increased in the embryo from 12 to 48 h, whereas *OsNOX7* and *9* expressions increased from 12 to 24 h and decreased thereafter. As expected, DPI inhibits the expression at predetermined time points for each of these genes. Taken together, these results suggest that ROS produced by NOXs are involved in radicle and root elongation during rice seed germination, and *OsNOX5*, *7* and *9* could play crucial roles in rice seed germination. These findings will facilitate further studies of the roles of ROS generated by NOXs during seed germination and seedling establishment and also provide valuable information for the regulation of NOX family gene expression in germinating seeds of monocot cereals.

## 1. Introduction

Seed germination is a complex physiological and biochemical process that involves signal transduction and regulation of gene expression [1,2,3,4,5]. Previous studies have shown that reactive oxygen species (ROS) are central components of plant adaptation to biotic and abiotic stresses and also function as signaling molecules to either positively or negatively regulate seed germination [3,6,7,8,9,10]. In seeds, ROS play roles in endosperm weakening, mobilization of seed reserves, protection against pathogens and programmed cell death [3,11].

Plasma-membrane NADPH oxidases (NOXs) are the key enzymes in plants for ROS generation [12]. They catalyze the production of the superoxide radical (O_2_^−^) by translocating an electron from NADPH inside the cell across the membrane to interact with molecular oxygen via flavin adenine dinucleotide. The O_2_^−^ then undergoes chemical transformation to yield hydrogen peroxide (H_2_O_2_) and O_2_. Peroxidases then catalyze the formation of ^•^OH from O_2_^−^ and H_2_O_2_ in the apoplast. Because ^•^OH is the ROS with the highest reactivity and shortest lifespan, it can directly cleave cell wall polysaccharides and thus loosen the plant cell wall of the seed endosperm cap in dicots such as cress [13,14]. A previous study has demonstrated that O_2_^−^, H_2_O_2_ and peroxidase (an alternative marker for ^•^OH production) specifically accumulate in the endosperm cap and radicle of lettuce seeds [15]. Thus, it may be logical to hypothesize that ROS generated by plasma membrane NOXs might also be involved in monocot seed germination.

NOXs are ubiquitous among fungi, animals and plants, but they are not found in prokaryotes and most unicellular eukaryotes [16]. We recently characterized the origin and evolution of plant NOX genes [17]. Their biological importance and physiological functions have also been characterized in several plant species. For example, eight ferric reduction oxidases (FROs; ancient NOXs; FRO1–8) and ten typical NOXs (regulatory burst oxidase homolog A–J, RBOHA–J) in *Arabidopsis* have been reported, and their functions vary with different isoforms during developmental stages such as root hair tip growth [18], lateral root growth [19], pollen tube tip growth [20,21], endosperm development [22] and seed after-ripening [23], and during different stress responses such as salt-stress tolerance [24] and disease resistance [25]. In contrast, only two FROs and nine typical NOXs genes have been identified in rice, and their expression profiles also show unique stress- and hormone-response characteristics [17,26,27,28]. Additionally, plant NOXs, as key signaling nodes, integrate a multitude of signal transduction pathways with ROS signaling such as the Ca^2+^-dependent protein kinase [29] and mitogen-activated protein kinase (MAPK) pathways [30] and hormone signal transduction cascades [30]. Moreover, *MAPK2* is activated and its expression greatly increased by exogenous H_2_O_2_, whereas no change is observed in the expression of *MAPK3* during seed germination of pea (*Pisum sativum*) [31]. Therefore, many previous studies indicate that different NOX homologs might play their specific roles during plant developmental processes and stress tolerance owing to the diversity of their functions. 

In dicot seeds (e.g., lettuce [32], tomato [33] and cress [14]), the endosperm has high mechanical strength and acts as a physical barrier to the completion of germination. Therefore, both endosperm cap weakening and radicle elongation are prerequisites for germination. These two events require cell wall loosening, which involves both enzymatic (e.g., endo-β-1,4-mannanases, pectin-methylesterases, cellulase) and non-enzymatic (e.g., ROS; including O_2_^−^, H_2_O_2_ and ^•^OH) mechanisms [34,35,36,37]. In contrast, the coleorhiza, a non-vascularized multicellular embryonic tissue, covers the seminal roots of monocot seeds and is thought to have a role in protecting the emerging root and, more recently, it has been also associated with the regulation of radicle emergence upon germination of monocot seeds, such as those of *Hordeum vulgare* and *Brachypodium distachyon* [38,39]. But whether the abovementioned enzymatic and non-enzymatic mechanisms also hold true in rice seed germination remains unknown. Because NOXs are key enzymes for ROS generation, some have been demonstrated to play essential roles during seed germination. For example, RBOHC, D and F in *Arabidopsis* play pivotal roles in root hair development and root elongation [40,41]. A research group has determined the subcellular localization of NOX mRNAs in barley seed using tissue printing, and they proposed the involvement of ROS produced by NOX in seed germination [42]. However, the relationships among ROS, NOXs and rice seed germination remain unclear. We thus investigated the morphology and germination percentage of rice seeds as well as the accumulation of O_2_^−^, H_2_O_2_ and ^•^OH during rice seed germination and seedling growth. Moreover, we performed a comprehensive analysis of NOX family gene expression profile during rice seed germination. The results broaden our understanding of the roles of rice NOXs in coleorhiza-limited seed germination and provide insight to further understand the physiological role(s) of the NOX family genes in plants.

## 2. Results

### 2.1. Diphenyleneiodinium (DPI) Inhibits Germination and Radicle Elongation of Rice Seeds

**Figure 1:**
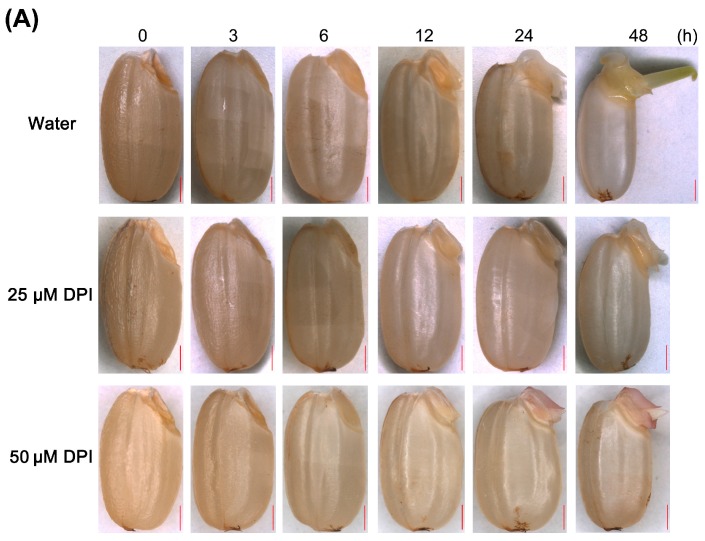

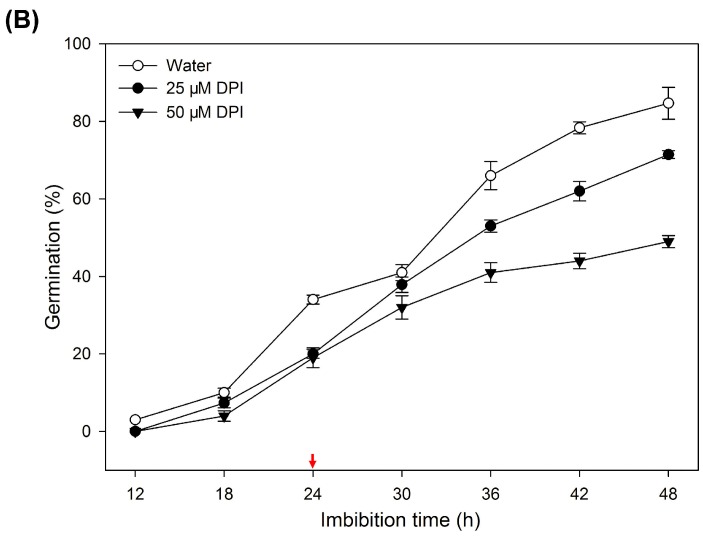
Morphologies and germination time courses of rice seeds in water and in the presence of diphenyleneiodinium (DPI). (**A**) The morphologies of rice seeds incubated in water or 25 μM DPI at different time points. The first seeds to complete germination *sensu stricto* were observed with the radicle protrusion from coleorhiza at 24 h. Scale bar, 1000 μm; (**B**) The germination time courses of rice seeds incubated in water or with DPI (25 or 50 μM). Germination was scored every 6 h for a total of 48 h, and the results are presented as the cumulative germination percentage. Data represent the mean ± SE of three biological replicates of 100 seeds each. The time point for the first seeds to complete germination *sensu stricto* was labeled with a red arrow.

To test if DPI affects rice seed germination, rice seeds without glume were incubated in water or in water containing the NOX inhibitor DPI (Figure 1A,B and Figure S1). The first seeds with coleorhiza that emerged were observed at 12 h after incubation, and followed by radicle protrusion from the coleorhiza at 24 h, which indicated complete germination *sensu stricto*. The final germination percentage increased rapidly in water and reached 41% at 30 h, and 84% at 48 h (Figure 1B). When incubated with 25 or 50 μM DPI, the time for the first seeds to complete germination was comparable to that of seeds in water at 24 h; however, the germination percentage decreased (Figure 1B), and the growth of the radicle and coleoptile were also inhibited (Figure 1A and Figure S1). Notably, germination was more strongly inhibited by 50 μM DPI than by 25 μM DPI, and the time at which 50% germination was achieved was delayed until 48 h and 36 h, respectively. After 48 h, 71% of seeds had completed germination in 25 μM DPI compared with only 49% in 50 μM DPI. This result indicated that DPI is a potent inhibitor of embryo growth, especially of radicle and root elongation, and its inhibitory effect was dose dependent. Therefore, 25 μM DPI was selected to further investigate the relationship between NOXs and rice seed germination.

### 2.2. Embryo Viability Decreases after 25 μM DPI Treatment

**Figure 2:**
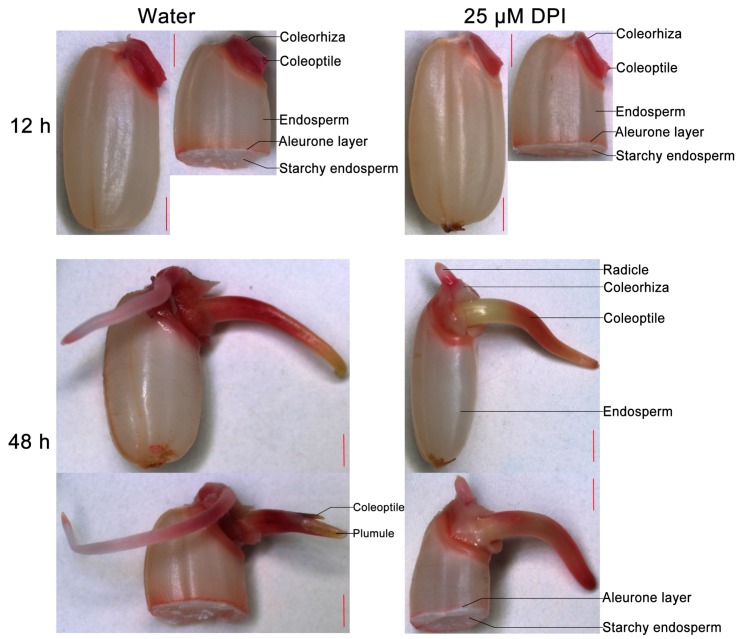
2,3,5-triphenyltetrazolium chloride (TTC) staining of rice seeds incubated in water or with 25 μM DPI for 12 or 48 h. After staining, the whole and half granule seeds with embryo were photographed, respectively. Dark-red staining indicates the strong viability of cells or tissues, while light-pink staining indicates the reduced viability of cell or tissue in germinating rice seed. Scale bar, 1000 μm.

To determine whether the viability of the embryo and endosperm is influenced by DPI, seeds incubated in water or with 25 µM DPI were stained with 2,3,5-triphenyltetrazolium chloride (TTC) and photographed (Figure 2). When seeds were germinated in water, the embryo at 12 h and coleorhiza, radicle and coleoptile at 48 h were all stained, whereas the starchy endosperm showed no staining. Interestingly, the aleurone layer of the whole seed showed negligible staining, which would be related with the uptake of reactive dye TTC. When the seed was cut in half, however, the aleurone layer was also stained. Moreover, when the seeds were incubated with 25 µM DPI, the staining of the embryo at 12 h and coleorhiza, radicle and coleoptile at 48 h was weaker than in water, and some tissues (e.g., zone of coleoptile near coleorhiza) were not stained. Remarkably, staining of the aleurone layer of half seeds was not dramatically inhibited by 25 µM DPI compared with seeds in water. These results demonstrated that DPI specifically reduced the viability of the embryo, especially the coleorhiza and radicle, but not that of the aleurone layer.

### 2.3. O_2_^−^ Is Produced and Accumulates in the Embryo of Seeds in Water, Especially in the Coleorhiza and Radicle, though This Process Is Inhibited by 25 µM DPI

**Figure 3:**
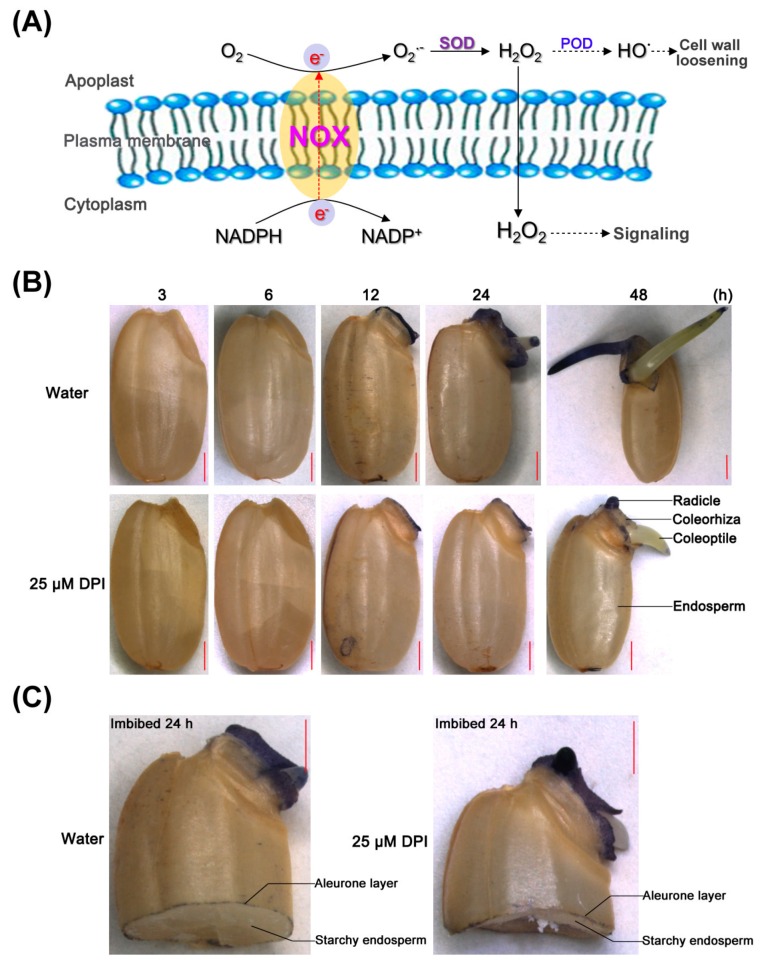

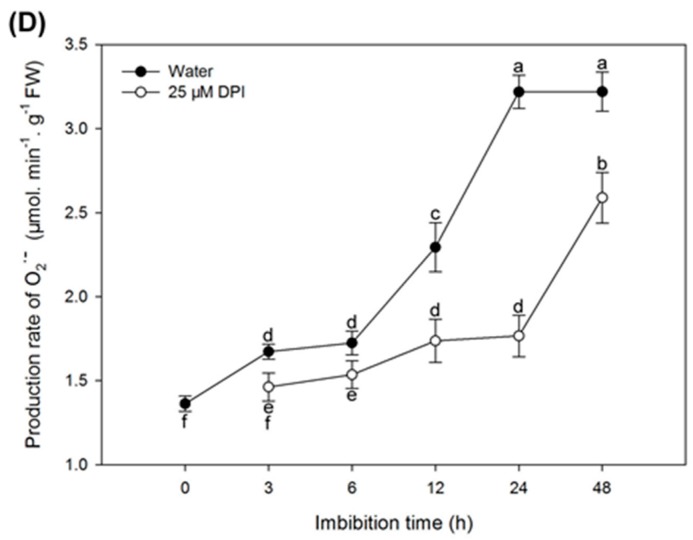
Histochemical staining and quantification of O_2_^−^ in rice seeds incubated in water or with DPI. (**A**) Schematic model for reactive oxygen species (ROS) generation by NADPH oxidases. SOD, superoxide dismutase. POD, peroxidase; (**B**,**C**) Histochemical staining by nitrobluetetrazolium (NBT) to determine the localization of O_2_^−^ in whole seeds incubated in water or with 25 μM DPI for 3, 6, 12, 24, 48 h and half granule seeds with embryo for 24 h, respectively. Scale bar, 1000 μm; (**D**) Quantification of the O_2_^−^ production rate in rice seeds incubated in water or with 25 μM DPI for 0, 3, 6, 12, 24 or 48 h. Data represent the mean ± SE of three biological replicates of 30 embryos (~0.1 g) each. Means denoted by the same letter do not significantly differ at *p* < 0.05 (Fisher’s least significant difference test). FW, fresh weight.

A previous study indicated that ROS in plant cell walls are primarily produced by plasma membrane-localized NOXs that reduce molecular oxygen to superoxide by oxidizing NADPH via flavin adenine dinucleotide and two heme groups (Figure 3A) [43]. The primary product, O_2_^−^, is then converted to H_2_O_2_ by superoxide dismutase. Finally, peroxidases catalyze the formation of ^•^OH from O_2_^−^ and H_2_O_2_ in the apoplast [13,44]. A portion of ROS participate in the regulation of respiratory metabolism, mobilization of nutrients and other physiological processes through signal transduction [45,46], whereas some ROS can directly cleave cell wall polysaccharides, further loosening the plant cell wall and eventually facilitating the completion of germination [47].

The production and accumulation of O_2_^−^ were investigated by histochemical nitrobluetetrazolium (NBT) staining of rice seeds incubated in water or 25 μM DPI. When seeds were incubated in water, the coleorhiza and radicle showed strong staining at 12 h, the time point of the first seed’s coleorhiza emergence, but no staining was observed from 3 to 6 h (Figure 3B). Interestingly, the coleoptile of the seeds showed no staining except at the tip, suggesting that O_2_^−^ may play a different role in protrusion of radicle and coleorhiza from that in the emergence of coleoptile. In addition, the aleurone layer of the whole seed was not stained (Figure 3B,C), but when the seed was crosscut at the middle of the endosperm, the aleurone layer was stained slightly although the starchy endosperm was still not (Figure 3C), similar to what has been seen for lettuce seed endosperm [15]. This might be due to either O_2_^−^ not being produced in outer cells of the aleurone layer or to wounding response or something similar in the tissue.

When seeds were incubated with 25 µM DPI, the intensity of staining in the embryo, as well as in the aleurone layer, was reduced, especially at the coleorhiza (Figure 3B,C). The rate of O_2_^−^ generation in the embryo was also quantified by colorimetry (Figure 3D). When the seeds were incubated in water, the rate in the embryo increased slowly up to 6 h, then rose sharply until 24 h, and remained high from 24 to 48 h. However, the rate was relatively low in the seed embryos in 25 µM DPI, increasing slowly up to 24 h and sharply upregulated after. The results of O_2_^−^ quantification were consistent with the histochemical staining.

### 2.4. H_2_O_2_ Is Produced and Accumulated in the Embryo and Aleurone Layer of Seeds in Water, but This Process Is Inhibited by 25 µM DPI Only in the Embryo

**Figure 4:**
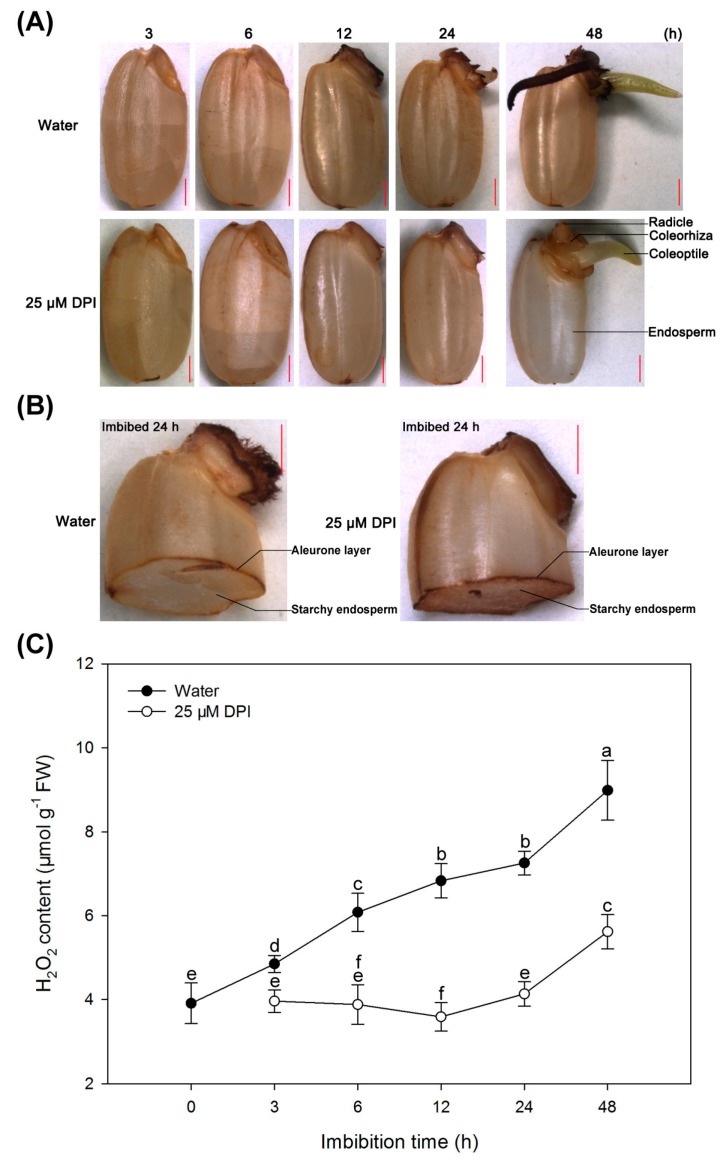
Histochemical staining and quantification of H_2_O_2_ in rice seeds in water or with DPI. (**A**,**B**) Histochemical staining with DAB to determine the localization of H_2_O_2_ in whole seeds incubated in water or with 25 μM DPI for 3, 6, 12, 24, 48 h and half granule seeds with embryo for 24 h, respectively. Scale bar, 1000 μm; (**C**) Quantification of H_2_O_2_ content in rice seeds incubated in water or with 25 μM DPI for 0, 3, 6, 12, 24 or 48 h. Data represent the mean ± SE of three biological replicates of 30 embryos (~0.1 g) each. Means denoted by the same letter do not significantly differ at *p* < 0.05 (Fisher’s least significant difference test). FW, fresh weight.

The production and accumulation of H_2_O_2_ were investigated by 3,3-diaminobenzidine hydrochloride (DAB) staining. As shown in Figure 4A, when seeds were incubated in water, the staining of the embryo was gradually increased during the whole imbibition process, and H_2_O_2_ was located mainly in the embryo before seed germination and in the coleorhiza and radicle of germinated seeds. Notably, the endosperm was also stained faintly, unlike with the NBT staining, suggesting that either DAB could penetrate the endosperm layer or H_2_O_2_ was produced in the outer cells of the aleurone layer. When the seeds were incubated with 25 µM DPI, the staining intensity of the embryo remained constant from 3 to 6 h. However, the staining intensity in the coleorhiza and radicle was reduced from 12 to 48 h, especially at 48 h, compared to seeds in water. Interestingly, staining of the aleurone layer of seeds was either not or less inhibited by 25 µM DPI, compared with seeds in water (Figure 4B).

The H_2_O_2_ content in the embryo was further quantified by colorimetry (Figure 4C). When seeds were incubated in water, the H_2_O_2_ content in the embryo increased progressively during the imbibition process and peaked at 48 h. However, the H_2_O_2_ content of embryo incubated in 25 µM DPI was significantly lower than that in water during the germination course, which was similar to the production rate of O_2_^−^. These results were in agreement with the histochemical staining.

### 2.5. Peroxidase Activity Increases in Both the Embryo and Aleurone Layer of Seeds in Water and Is Inhibited by 25 µM DPI

**Figure 5:**
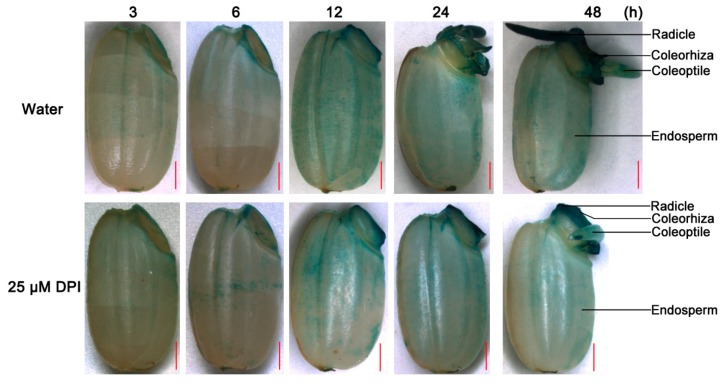
Histochemical detection of peroxidase activity in rice seeds incubated in water or with DPI. Histochemical staining by TMB for the localization of peroxidase activity detected in whole seeds incubated in water or with 25 μM DPI for 3, 6, 12, 24 and 48 h. Scale bar, 1000 μm.

As ^•^OH has the shortest lifespan among ROS, it is difficult to quantify directly [14]. Hence, we assayed peroxidase activity, which serves as an approximate indirect measurement of ^•^OH production [15]. Peroxidase activity in rice seeds was determined by TMB staining. In general, when rice seeds were imbibed in water or 25 µM DPI, the staining intensity of embryo and endosperm by TMB were stronger than by NBT and DAB. As shown in Figure 5, unlike the O_2_^−^ and H_2_O_2_ staining, the endosperm showed visible staining. Meanwhile, the intensity of TMB staining in the embryo increased with imbibition time, especially in the coleorhiza and radicle. Notably, the coleoptile was faintly stained, similar to what was observed for O_2_^−^ and H_2_O_2_, suggesting that ROS may be more important for coleorhiza and radicle protrusion than coleoptile emergence. When seeds were incubated with 25 µM DPI, the intensity of TMB staining in the endosperm and embryo (especially in the coleorhiza) was decreased (Figure 5) relative to seeds in water, indicating that DPI reduced the peroxidase activity of germinating seeds.

### 2.6. Reactive Oxygen Species (ROS) Are Crucial for Radicle and Root Elongation but Not for Seedling Leaf Growth

**Figure 6:**
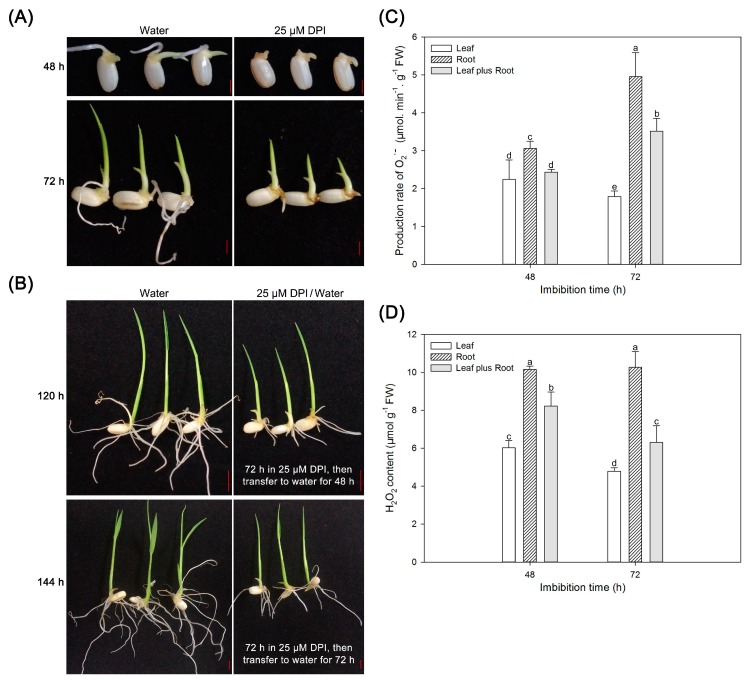
Seedling morphologies and quantification of O_2_ˉ and H_2_O_2_ in leaves and root of rice seedlings. (**A**) Morphology of seedlings from rice seeds germinated in water or with 25 μM DPI for 48 and 72 h. Scale bar, 2000 μm; (**B**) Morphology of seedlings from rice seeds germinated in water for 120 h (**left**, **upper**) and 144 h (**left**, **lower**) or first germinated in the presence of 25 μM DPI for 72 h and then transferred to water for 48 h (**right**, **upper**) and 72 h (**right**, **lower**). Scale bar, 5000 μm; (**C**,**D**) Quantification of O_2_ˉ and H_2_O_2_ in leaves, root and leaves plus root of rice seedlings incubated in water for 48 and 72 h. Data represent the mean ± SE of three biological replicates of 30 embryos at 48 h and 15 embryos at 72 h (0.2–0.3 g total). Means denoted by the same letter do not significantly differ at *p* < 0.05 (Fisher’s least significant difference test).

The germination percentage and phenotype of germinating seeds revealed that DPI inhibited radicle elongation. As such, we further investigated whether DPI could also inhibit root growth and development. As shown in Figure 6A and Figure S2, when the seeds were incubated in water for 48 or 72 h, the seminal root elongated rapidly, indicating a strong growth potential. When the seeds were incubated with 25 µM DPI, however, the emergence of the radicle (at 48 h) and elongation of the root (at 72 h) were dramatically suppressed, and roots, if present, were short. Interestingly, the growth of the coleoptile and seedling leaves was barely inhibited by DPI, indicating that the radicle and seminal roots are more sensitive to DPI than the coleoptile and seedling leaves. Moreover, when the seedlings were incubated with 25 µM DPI for 72 h and then transferred to water for 48 or 72 h, the growth of seminal roots recovered to some extent (Figure 6B and Figure S2). Additionally, the rates of O_2_^−^ and H_2_O_2_ production in seedlings (leaf plus root), roots and leaves were further investigated (Figure 6C,D). For whole seedlings cultured in water, O_2_^−^ production increased significantly from 48 to 72 h, while the H_2_O_2_ content decreased significantly. In the leaves of the seedlings, O_2_^−^ production and H_2_O_2_ content decreased from 48 to 72 h. In contrast, in the roots, O_2_^−^ production increased markedly and H_2_O_2_ content remained at a high level when seedlings were incubated in water. These results might suggest that O_2_^−^ and H_2_O_2_ are more important for root growth than for leaf sprouting during seedling establishment, similar to the results for radicle and coleoptile of seeds during rice germination (Figure 3, Figure 4 and Figure 5).

### 2.7. Different Expression Profiles of NOX Family Genes during Rice Seed Germination

**Figure 7:**
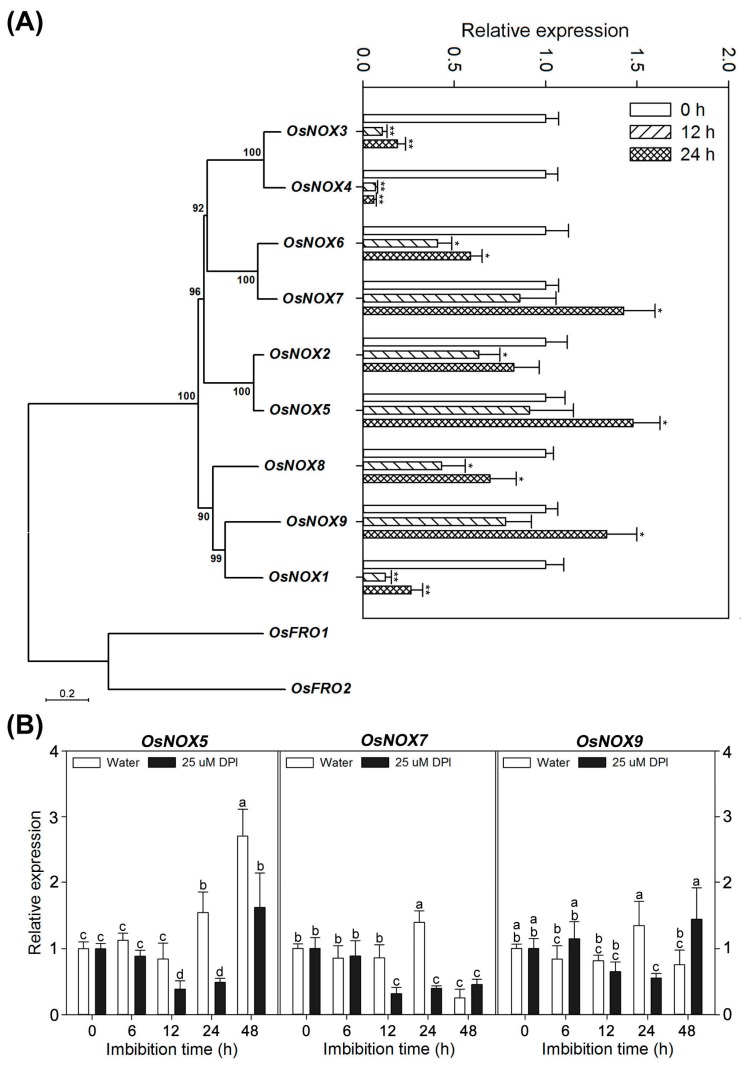
Expression profiles of the typical NOX genes *OsNOX1–9* during germination of rice seeds. (**A**) Phylogenetic relationships of rice NOX family genes and expression profiles of the typical NOX genes (*OsNOX1–9*, [17]) in embryos assayed by qPCR after seeds had been incubated in water for 0, 12 and 24 h. Data represent the mean ± SE of three biological replicates of 30 embryos (~0.1 g). Significant differences for the qPCR data at 12 and 24 h from those at 0 h were assessed by the Student’s *t*-test (* *p* < 0.05; ** *p* < 0.01); (**B**) Expression of *OsNOX5*, *7* and *9* in the embryo assayed by qPCR after seed imbibition in water or with 25 µM DPI for 0, 6, 12, 24 or 48 h. Data represent the mean ± SE of three biological replicates of 30 embryos (~0.1 g). Means denoted by the same letter do not significantly differ at *p* < 0.05 (Fisher’s least significant difference test).

To select the candidate gene(s) that best reflected NOX function and activity during seed germination, qPCR was performed to assess the expression of *OsNOX1–9* in germinating rice seeds in water for 0, 12 and 24 h (Figure 7A). When seeds were incubated in water, the transcriptions of *OsNOX1*, *3* and *4* were sharply downregulated, whereas *OsNOX2*, *6* and *8* were slightly or clearly decreased at levels comparable with those in dry seeds, although the abundance of some transcripts increased slightly from 12 to 24 h. In contrast, *OsNOX5*, *7* and *9* were expressed relatively higher when seeds were in water for 24 h, and the expression levels of these three genes were consistent with the changes in germination percentage and accumulation of ROS of germinating seed (Figure 1B and Figure 3, Figure 4 and Figure 5). Therefore, *OsNOX5*, *7* and *9* were chosen as candidate genes that could possibly account for most NOX expression and activity during rice seed germination.

The expression of *OsNOX5*, *7* and *9* was then examined throughout the course of seed germination (at 0, 6, 12, 24 and 48 h) by qPCR (Figure 7B). For seeds incubated in water, expression of the three genes remained essentially unchanged before 12 h (time point of the first coleorhiza emergence). When seeds were incubated in water from 12 to 48 h (emergence of the radicle and coleoptile elongation; Figure 1 and Figure 6A), transcription of *OsNOX5* progressively increased in the embryo, whereas the expression of *OsNOX7* and *9* increased from 12 to 24 h and decreased thereafter. As mentioned earlier, DPI is a potent ROS inhibitor [48], but whether *OsNOX5*, *7* and/or *9* are also inhibited by this reagent required further investigation. As shown in Figure 7B, for seeds incubated with 25 µM DPI, expression of the three genes in the embryo was similar to that of seeds in water before 6 h. When seeds were incubated with 25 µM DPI for 12 to 48 h, transcription of *OsNOX5* was repressed significantly, whereas that of *OsNOX7* and *9* decreased markedly at some time point (i.e., 24 h, when the first radicles protrude from the coleorhiza). The differences in expression profiles of *OsNOX5*, *7* and *9* in the embryo between seeds incubated in water and DPI suggest diverse roles in radicle and root elongation.

## 3. Discussion

### 3.1. Difference in Germination Characteristics between Dicots and Monocots: Involvement of ROS Generated by NOXs in Rice Seed Germination and Root Elongation of Seedling

By definition, germination starts with the uptake of water by the quiescent dry seed and terminates with the elongation of the embryonic axis and the protrusion of the radicle from its enclosure [31,45]. For seeds of dicots (e.g., lettuce, cress and tomato), the endosperm has a high mechanical strength and acts as a physical barrier to the completion of germination [36,49,50]. For these seeds, micropylar endosperm weakening and radicle elongation are essential for the germination process. However, for seeds of monocots, such as rice, barley, wheat and *Brachypodium distachyon*, the embryo is located on the side of the endosperm, and the endosperm is thought to provide nutrition for seed germination and seedling growth [45]. During rice seed germination (*sensu stricto*) and seedling establishment, the epiblast and coleorhiza appeared first, followed by the emergence of the coleoptile, and emergence of radicle from the coleorhiza, and then the primary leaf (also called the first seedling leaf) from the coleoptile (Figure 1A and Figure 6A). Although the coleorhiza is considered a key tissue protecting root emergence in dormant barley seeds, it has been recently thought to have a role in preventing the emerging root during seed germination, which is similar to what has been proposed for the endosperm of dicot seeds [38,42,51]. In this study, the results suggested that the coleorhiza in rice seeds may serve as a barrier for radicle protrusion to complete germination (*sensu stricto*), and thus it appears to have a similar function as the micropylar endosperm in dicot seeds. In fact, we also observed the coleorhiza was gradually “dissolved” with the process of rice seed germination, especially the imbibition after 6 h (unpublished data). This is also similar to what has recently been reported in other monocots, namely barley and *Brachypodium distachyon* [39,42,51].

Many studies have demonstrated that both endosperm weakening and radicle elongation during germination require cell wall loosening, which involves both enzymatic and non-enzymatic mechanisms [34]. As for the enzymatic mechanisms involved in seed germination, endo-β-mannanase, cellulase, pectin methylesterase and α-galactosidase have been found in the endosperm cap and radicle during germination of lettuce, tomato and cress seeds [36,49,50,52]. β-galactosidases and endo-β-mannanase have also been detected in the coleorhiza, radicle and aleurone layer of rice seeds, and they are likely to be involved in rice seed germination [53,54]. However, relatively little has been reported about the relationship between non-enzymatic mechanisms (e.g., ROS) and seed germination. Although previous studies have traditionally emphasized the deleterious effects of ROS in seed biology [55], the more recent studies have reported that O_2_^−^, H_2_O_2_ and ^•^OH are located in the endosperm cap and radicle of germinating lettuce seeds, and verified that ROS play positive roles in endosperm weakening and radicle elongation during lettuce seed germination [15]. As the cell wall in plants is mainly composed of polysaccharide molecules, ROS may directly cleave wall polysaccharides to help weaken the integrity of the cell wall and facilitate completion of germination [13,14,47]. In this study, we also found that production and accumulation of O_2_^−^, H_2_O_2_ and ^•^OH gradually increased in the embryo of rice seeds in parallel with an increase in the germination percentage, and the levels of these three types of ROS were higher in the coleorhiza and radicle than in the coleoptile of germinating rice seeds (Figure 3, Figure 4 and Figure 5). These results indicate that the non-enzymatic reactions—specifically those involving ROS—might be required in rice seed germination. The involvement of ROS in the loosening of cell walls of the coleorhiza and radicle in rice seeds, which was found in lettuce seeds [15], needs further investigation. Interestingly, production and accumulation of O_2_^−^ and H_2_O_2_ in roots were remarkably greater than those in leaves (Figure 6C,D), indicating that ROS might be more crucial for root elongation than leaf growth during rice seedling establishment. Although O_2_^−^ can be found in all the regions of radicle and coleorhiza, it was only detected in the tip region of the coleoptile, suggesting that O_2_^−^ may play different roles in protrusion of radicle and coleorhiza compared with emergence of coleoptile (Figure 3B).

Moreover, there are many pathways for ROS generation in plant cells. We previously showed that H_2_O_2_ produced by polyamine oxidase is likely involved in rice seed germination [37]. In this study, we focused on NOXs, a major enzymatic route of ROS synthesis in plant cells. We examined the relationships among NOX-produced ROS, rice seed germination and seedling establishment. As NOX is a kind of protein localized in cell membrane, it is difficult to assay its enzyme activity. Hence, we block the activity of the NOX with DPI, a specific inhibitor of NOX [42,48], to investigate the relationship between ROS and rice seed germination from another perspective. As shown in Figure 1 and Figure 6A,B, DPI inhibited the process of rice seed germination and seedling growth, especially the elongation of radicle and roots. These are in accordance with the decrease of ROS production in these tissues (Figure 3, Figure 4 and Figure 5 and Figure 6C,D), indicating that NOXs, as well as their catalytic products ROS, play an essential role in radicle emergence and root growth. These findings were similar to a previous observation that inhibition of NOX delayed germination and root growth but not coleoptile growth during switchgrass seed germination [41]. In addition to O_2_^−^, production and accumulation of H_2_O_2_ and ^•^OH also decreased gradually in embryos of seeds upon incubation with DPI (Figure 3, Figure 4 and Figure 5). This may be because O_2_^−^ generated by NOX occurs upstream in the ROS production chain, and thus the O_2_^−^ content may directly determine the production of H_2_O_2_ and ^•^OH. Although radicle and root growth of germinating rice seeds were inhibited by DPI, they could be partially recovered by transferring the seeds incubated with DPI to water (Figure 6A,B). It may be that NOX activity, and therefore ROS production, was regained in water.

### 3.2. Functional Diversity and the Possible Role(s) of the Rice NOX Family in Rice Seed Germination and Root Elongation

As mentioned earlier, NOXs, by producing ROS, play important roles in seed germination. However, different characteristics among the NOXs suggest functional diversity. We previously identified 11 NOX homologs in rice, which were functionally classified into ancient (*OsFRO1* and *2*) and typical (*OsNOX1–9*) types and subdivided into four conserved subfamilies [17]. Moreover, the expression patterns at different developmental stages and under various abiotic stresses and hormone treatments differed among the NOXs [17,27,28], suggesting functional diversity of NOXs during development and in response to stress and hormones. These findings are similar to what has been reported for the *Arabidopsis* RBOH family [12].

For example, the *atrbohC* mutant has defects in root hair development, and *atrbohD/F* double and *atrbohF* single mutants are less susceptible to abscisic acid-mediated inhibition of root elongation [56]. Moreover, a recent report demonstrated that NOX mRNAs are expressed in the embryo and aleurone cells of barley seeds, and these expression sites are consistent with the sites of ROS production in the seeds after imbibition [42]. In our present study, we also found that the expression levels differed among the nine typical NOX genes, suggesting functional diversity during rice seed germination (Figure 7). Interestingly, the transcription of *OsNOX1*, *3* and *4* were significantly downregulated in embryos of imbibed seeds before 24 h compared with dry seeds (Figure 7A), not comparable to the changes in germination percentage and ROS production during rice seed germination (Figure 1B and Figure 3, Figure 4 and Figure 5). This suggests that *OsNOX 1*, *3* and *4* are the genes most unlikely involved in rice seed germination. Furthermore, transcription of several other genes, especially *OsNOX5*, *7* and *9*, increased gradually and peaked at 24 h for *OsNOX7* and *9*, in accordance with the changes in the accumulation of O_2_^−^, H_2_O_2_ and ^•^OH as well as germination percentage. This suggests that these three genes may play a crucial role in radicle elongation during rice seed germination. In addition, when the seeds were incubated in 25 μM DPI, the transcription of *OsNOX5* was significantly inhibited from 12 to 48 h, while *OsNOX7* from 12 to 24 h and *OsNOX9* only at 24 h (Figure 7B). Combined with the germinating morphology, germination percentage and ROS production results, this finding suggests that *OsNOX7* and *9* are involved in seed germination *sensu stricto*, whereas *OsNOX5* may be essential in seed germination *sensu stricto* as well as seedling establishment. Of course, further experiments were needed to validate the roles of *OsNOX5, 7* and *9* on the regulation of rice seed germination by utilizing single mutants or gene knockout methods.

## 4. Experimental Section

### 4.1. Non-Plant Materials

Diphenyleneiodinium (DPI), nitrobluetetrazolium (NBT), 3,3-diaminobenzidine hydrochloride (DAB), 3,3′,5,5′-tetramethylbenzidine (TMB) and 2,3,5-triphenyltetrazolium chloride (TTC) were purchased from Sigma-Aldrich (St. Louis, MO, USA). Water used was always doubly distilled.

### 4.2. Plant Materials and Seed Germination

Rice seeds (*Oryza sativa* ssp. *japonica* cv. Nipponbare) with the glume removed were placed in a transparent plastic germination box (12 × 12 × 6 cm^3^) containing two layers of filter paper and 20 mL of water or 25 μM DPI in water. The germination boxes were incubated in a growth chamber at 28 ± 1 °C under a 16 h light (10,000 lux)/8 h dark photocycle and the germination tests were started at light. The seeds with protruded radicles were regarded as having completed germination and counted to calculate the germination rate every 6 h for a total of 48 h. Seeds were photographed using a Zeiss SteReo Lumar V12 compound stereomicroscope (Carl Zeiss, Jena, Germany).

### 4.3. 2,3,5-Triphenyltetrazolium Chloride (TTC) Staining

TTC staining is a frequently used method to determine the viability of cells or tissues in animals and plants. TTC is a white salt and substrate of dehydrogenases and its metabolite is a red dye, which turns the cell red in viable tissue and white in dead tissue. For TTC staining, 0.5 grams of TTC were dissolved in 2 mL alcohol, then dissolved to 100 mL of double distilled water and stored at 4 °C in the dark. After incubation in water or 25 μM DPI for 12 and 48 h, five whole seeds and five half granule seeds with embryo were selected and stained in 0.5% TTC at 35 °C for 3 h and then washed three times with water and photographed as described above.

### 4.4. Histochemical Localization and Quantification of O_2_^−^

Histochemical localization and quantification of O_2_^−^ were carried out with NBT staining and colorimetrical measurement, respectively, as described in our previous study [15]. For NBT staining, after the rice seeds were incubated in water or 25 μM DPI for 3, 6, 12, 24 or 48 h, five whole seeds and five half granule seeds with embryo were selected and incubated in 1 mM NBT in 10 mM Tris-HCl (pH 7.0) at room temperature for 30 min and then washed in water and photographed as described above. For quantification of O_2_^−^, the production rate of O_2_^−^ (nmol O_2_^−^ min^−1^·g^−1^ fresh weight) was measured by a colorimetrical method, for which 30 embryos from each of the abovementioned five time points were extracted in 2 mL of 50 mM phosphate buffer containing 1 mM EDTA, 0.3% (*w*/*v*) Triton X-100 and 2% polyvinyl pyrrolidone (pH 7.8). The samples were homogenized by Tissuelyser-24 at 4 °C and then the homogenate was centrifuged at 12,000× *g* for 20 min. A 1 mL aliquot of supernatant solution was mixed with 1 mL of 50 mM phosphate buffer (pH 7.8) and 1 mL of 1 mM hydroxylamine hydrochloride and incubated at 25 °C for 1 h. After adding 1 mL of 17 mM *p*-amino benzene sulfonic acid and 1 mL of 7 mM α-naphthylamine, the mixture was incubated at 25 °C for 20 min. Absorbance was then read at 530 nm. A standard response curve was prepared with a known concentration of NO_2_^−^ using the same method as described above.

### 4.5. Histochemical Localization and Quantification of H_2_O_2_

Quantification and histochemical localization of H_2_O_2_ were carried out by DAB staining and colorimetrical measurement, respectively, also as described in our previous study [15,36]. For DAB staining, after the rice seeds were incubated in water or with 25 μM DPI for 3, 6, 12, 24 or 48 h, five whole seeds and five half granule seeds with embryo were selected and incubated in 1 mg/mL DAB-HCl (pH 3.8) at room temperature for 30 min and then washed in water and photographed as described above. For quantification, H_2_O_2_ was extracted by the homogenization of 30 embryos from each of the indicated time points in 3 mL of cold acetone. The samples were homogenized by Tissuelyser-24 at 4 °C and then the homogenate was centrifuged at 12,000 rpm for 15 min. A 1 mL aliquot of supernatant solution was mixed with 0.1 mL of 5% titanium sulfate in concentrated HCl followed by the addition of 0.2 mL aqueous NH_3_ (25%) to precipitate the peroxide–titanium complex. The mixture was then centrifuged at 12,000× *g* for 15 min. The precipitate was solubilized in 3 mL of 2 mM H_2_SO_4_. Absorbance was then read at 415 nm. A standard response curve was prepared with a known concentration of H_2_O_2_ using the same method as described above.

### 4.6. Histochemical Detection of Peroxidase Activity

Peroxidase is a key enzyme in the production of ^•^OH, and its activity can indirectly reflect the production and accumulation of ^•^OH. Peroxidase activity was detected histochemically by TMB staining as described in our previous study [15,36]. For TMB staining, after the rice seeds were incubated in water or 25 μM DPI for 3, 6, 12, 24 or 48 h, five whole seeds and five half granule seeds with embryo were selected and incubated in 0.2% (*w*/*v*) TMB and 1 mM H_2_O_2_ in 20 mM phosphate buffer (pH 6.5) at room temperature for 30 min and then washed in water and photographed as described above.

### 4.7. Quantitative Real-Time PCR (qPCR)

For the expression profile analysis of the typical *OsNOX1*–9 genes [17] during the entire process of seed germination by qPCR, 30 embryos from each of the aforementioned five time points were homogenized and immediately frozen at −80 °C. Total RNA was extracted using the Column Plant RNAout 2.0 kit (TIANDZ, Beijing, China), and the subsequent qPCR analysis was as described in our previous studies [17,57]. All gene-specific primers were designed to span introns or cross an exon-exon junction whenever possible and avoid the conserved region. Primer sequences are shown in Table S1. The gene *OsGAPDH1* (RAP-DB ID: Os02g0601300) was chosen as the internal control.

### 4.8. Statistical Analysis

One-way analysis of variance was used to compare mean values, and when significant *p*-values were obtained (*p* < 0.05), differences between individual means were compared with the Fisher’s least significant difference test (*p* < 0.05). Student’s *t*-tests (* *p* < 0.05, ** *p* < 0.01) were conducted to evaluate variances in expression levels of *OsNOX1–9*.

## 5. Conclusions

In summary, the roles of reactive oxygen species (ROS) involved in seed germination appear to be similar in monocots and dicots. The coleorhiza in rice seeds appears to play a role similar to that of the micropylar endosperm of dicot seeds [15,58], and we propose that coleorhiza weakening and radicle elongation maybe prerequisite for monocot seeds germination (*sensu stricto*). Moreover, the observed changes in germination percentage and in superoxide radicals (O_2_^−^), hydrogen peroxide (H_2_O_2_) and hydroxyl radicals (^•^OH) generated by NOXs in germinating rice seeds and seedlings suggest that ROS produced by NOXs are involved in radicle and root elongation during rice seed germination. The expression of the NOX genes indicates that *OsNOX5*, *7* and *9* could play crucial roles in rice seed germination. Future studies will focus on further delineating the molecular functions of *OsNOX5*, *7* and *9* and the involvement of ROS produced by these three enzymes during rice seed germination.

## Supplementary Materials

Supplementary materials can be found at www.mdpi.com/1422-0067/18/1/110/s1.
