# Supplementary material for: Reactive Oxygen Species Generated by NADPH Oxidases Promote Radicle Protrusion and Root Elongation during Rice Seed Germination
Source: Int J Mol Sci. 2017 Jan 13;18(1):110. doi: 10.3390/ijms18010110 (PMC5297744; doi:10.3390/ijms18010110)
Supplement: Supplementary file 1 [file ijms-18-00110-s001.pdf]

Supplementary Materials: Reactive Oxygen Species Generated by NADPH Oxidases Promote Radicle Protrusion and Root Elongation during Rice Seed Germination

Wen-Yan Li, Bing-Xian Chen, Zhong-Jian Chen, Yin-Tao Gao, Zhuang Chen and Jun Liu

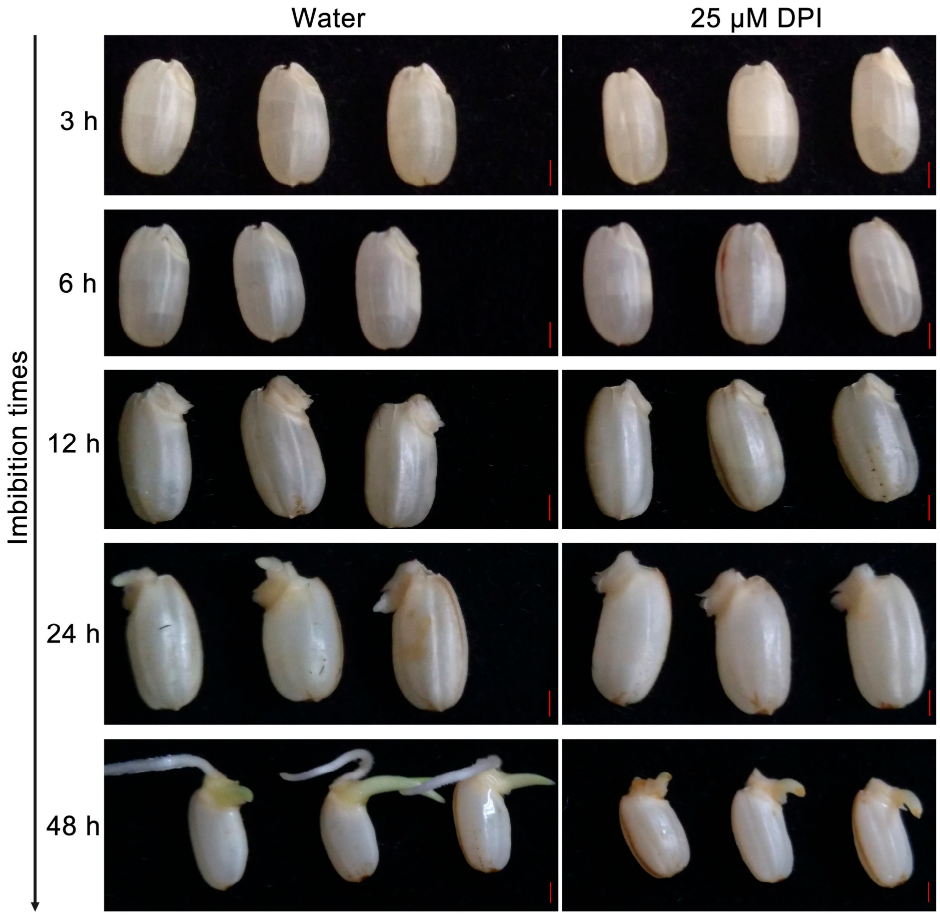

Figure S1. Germination morphologies of rice seeds incubated in water or with 25 μM DPI at different time points. Scar bar, 1000 μm.

Table S1. The gene-specific primers used for qRT-PCR in this study.

Gene Name	Primer Sequences	Amplicon Length	Span Introns/UTRs &
OsNOX1	Forward: 5′-GGCTTCAATGCCTTCTGGT-3′<br>Reverse: 5′-ATGGCTCCTAAACAACCGA-3′	180 bp	Intron 6
OsNOX2	Forward: 5′-ATCCGCAAAATAAGCACCTCT-3′<br>Reverse: 5′-CAGTAGCCCATCACATCAAAGAC-3′	155 bp	/
OsNOX3	Forward: 5′-TCAAGGCAGCGATTTACCC-3′<br>Reverse: 5′-CTCGCAAGCCTTCCCAAA-3′	233 bp	Introns 6, 7, 8
OsNOX4	Forward: 5′-CACAAGGTTATCGCACTGACG-3′<br>Reverse: 5′-AGCGATGAGTATGTTGGTTGA-3′	158 bp	Intron 5
OsNOX5	Forward: 5′-CCAGTGGGTGGGAAAAGTG-3′<br>Reverse: 5′-GTCCGATTGGCGGGTAAA-3′	247 bp	Introns 9, 10
OsNOX6	Forward: 5′-CCTTTCTCCATCACTTCAGCA-3′<br>Reverse: 5′-GGGCCATCTACAAGCAACC-3′	194 bp	Intron 8
OsNOX7	Forward: 5′-GTCAAATGCTTATGCTGTCA-3′<br>Reverse: 5′-TGTCCAGTCTCCGTTTGTT-3′	223 bp	Introns 7, 8
OsNOX8	Forward: 5′-GAGCGTGAGGCCAGTGAAGATA-3′<br>Reverse: 5′-AAGCTTGGGTTGGTCATGGCG-3′	330 bp	Introns 6, 7, 8
OsNOX9	Forward: 5′-TACTTCGGGCAGACACGGAT-3′<br>Reverse: 5′-GCGGGTTGCTGTCACTAAG-3′	151 bp	/
OsGAPDH1 *	Forward: 5′-GCAATCAAGGAGGAGGCTGA-3′<br>Reverse: 5′-ACGTGTCGCTCAAAGCAATG-3′	139 bp	Introns 8, 9

* *OsGAPDH1* (RAP-DB ID: Os02g0601300) was chosen as an internal control in rice; & UTRs, untranslated regions.
